# Soil Particles and Phenanthrene Interact in Defining the Metabolic Profile of *Pseudomonas putida* G7: A Vibrational Spectroscopy Approach

**DOI:** 10.3389/fmicb.2018.02999

**Published:** 2018-12-04

**Authors:** Andrea Fanesi, Asfaw Zegeye, Christian Mustin, Aurélie Cébron

**Affiliations:** Laboratoire Interdisciplinaire des Environnements Continentaux, CNRS, Université de Lorraine, Nancy, France

**Keywords:** bacteria, FTIR spectroscopy, FT-Raman spectroscopy, metabolic profile, multivariate classification analysis, phenanthrene, soil particles

## Abstract

In soil, organic matter and mineral particles (soil particles; SPs) strongly influence the bio-available fraction of organic pollutants, such as polycyclic aromatic hydrocarbons (PAHs), and the metabolic activity of bacteria. However, the effect of SPs as well as comparative approaches to discriminate the metabolic responses to PAHs from those to simple carbon sources are seldom considered in mineralization experiments, limiting our knowledge concerning the dynamics of contaminants in soil. In this study, the metabolic profile of a model PAH-degrading bacterium, *Pseudomonas putida* G7, grown in the absence and presence of different SPs (i.e., sand, clays and humic acids), using either phenanthrene or glucose as the sole carbon and energy source, was characterized using vibrational spectroscopy (i.e., FT-Raman and FT-IR spectroscopy) and multivariate classification analysis (i.e., PLS-DA). The different type of SPs specifically altered the metabolic profile of *P. putida*, especially in combination with phenanthrene. In comparison to the cells grown in the absence of SPs, sand induced no remarkable change in the metabolic profile of the cells, whereas clays and humic acids affected it the most, as revealed by the higher discriminative accuracy (*R*^2^, RMSEP and sensitivity) of the PLS-DA for those conditions. With respect to the carbon-source (phenanthrene vs. glucose), no effect on the metabolic profile was evident in the absence of SPs or in the presence of sand. On the other hand, with clays and humic acids, more pronounced spectral clusters between cells grown on glucose or on phenanthrene were evident, suggesting that these SPs modify the way cells access and metabolize PAHs. The macromolecular changes regarded mainly protein secondary structures (a shift from α-helices to β-sheets), amino acid levels, nucleic acid conformation and cell wall carbohydrates. Our results provide new interesting evidences that SPs specifically interact with PAHs in defining bacteria metabolic profiles and further emphasize the importance of studying the interaction of bacteria with their surrounding matrix to deeply understand PAHs degradation in soils.

## Introduction

Polycyclic aromatic hydrocarbons (PAHs) are persistent organic pollutants commonly found in industrially contaminated soils (Haritash and Kaushik, 2009). Differently from natural carbon (C) sources, the high hydrophobicity and low solubility in water of PAHs result in their adsorption to soil particles (SPs; here considered as mineral and organic components constituting the soil matrix), which is believed to limit the amount of C readily available for microbial growth (Weissenfels et al., 1992; Wilcke et al., 1996). Bacteria strategies to tackle low accessibility and availability of PAHs range from the direct contact with crystals or SPs, to the excretion of surfactants and to the expression of high affinity uptake systems (Miyata et al., 2004). This in turn allows the cells to maintain growth and cell viability even when PAHs are the sole C/energy source present.

The efficiency of bacteria in metabolizing PAHs is generally tested via mineralization assays. Although in the literature plenty of studies report such results, only a few have investigated how SPs alter bacteria ability to metabolize PAHs and how this affects the cell physiology (i.e., metabolic activity and metabolic profile). Limited experimental results suggested that rather than limiting cell activity, some SPs can enhance mineralization efficiencies by favoring bacterial contact with the adsorbed PAHs molecules (Ortega-Calvo and Saiz-Jimenez, 1998; Amellal et al., 2001) or by acting as surfactants. From a metabolic point of view, PAHs activate specific cellular pathways aimed at their metabolization and/or at the detoxification of resulting secondary metabolites. For instance, proteomic studies reported an up-regulation of cell components involved in PAHs metabolism (e.g., mono and di-oxygenases), in oxidative stress, cell energetics and C-metabolism (Seo et al., 2009 and references therein). Considering that specific SPs stimulate cells to express at their surface or excrete macromolecules involved in substrate attachment and in biofilm formation (Ojeda et al., 2008; Wu et al., 2014a,b,c), the interaction of PAHs and SPs may result in a complex reorganization of bacteria metabolic profile (i.e., cell macromolecular composition). Indeed, a global modification of bacterial transcriptome in the presence of particles and/or pollutant, was previously observed (Moreno-Forero and Van Der Meer, 2015; Lima-Morales et al., 2016).

The energy required for macromolecule synthesis in a given environmental niche is a fundamental limit for microorganism growth and colonization (VanBriesen, 2001; McCarty, 2007; LaRowe and Amend, 2016). Therefore, understanding how cells adjust their metabolic profile as a function of different SPs is critical to better understand spatial distributions of degraders and PAHs mineralization *in situ*, which could help identifying hotspots (i.e., location of high microbial activity) in soil (Kuzyakov and Blagodatskaya, 2015). An experimental approach accounting for SPs would therefore allow a more realistic understanding, with respect to the use of simple mineral media, of how bacteria cells metabolically react to the presence of PAHs in nature (Keum et al., 2008). Nevertheless, the complex interaction of bacteria with SPs has seldom been considered in experimental approaches regarding PAHs effect on cell metabolic profiles. In this study we therefore aimed at investigating how SPs influence the metabolic profile of bacteria during PAHs degradation.

In soil, the different SPs, such as sand, clays and humic acids, are responsible for binding different amounts of PAHs (Ortega-Calvo and Saiz-Jimenez, 1998; Uyttebroek et al., 2006), and to specifically modify the physico-chemical characteristics of the micro-environment in their immediate surroundings (Chenu, 1993; Vandevivere and Kirchman, 1993) leading to a highly heterogeneous environment. For instance, clays and humic acids have been reported to adsorb the highest fraction of PAHs, but to increase bacteria mineralization rates (Ortega-Calvo and Saiz-Jimenez, 1998; Uyttebroek et al., 2006). On the other hand, as presenting the lowest specific surface area cation exchange capacity and strength of binding sites, sand particles retain PAHs molecules the least and support low mineralization rates (Wilcke et al., 1996; Amellal et al., 2001; Müller et al., 2007; Uyttebroek et al., 2006). We hypothesized that for a specific bacteria species growing in the presence of different SPs, there is a specific metabolic profile related to each type of SPs, which reflects the distinctive micro-environmental conditions the cells are subjected to. In laboratory cultures where cells are grown in a liquid medium and SPs are suspended in it, this may hold true irrespective of whether the cells are attached to soil components or conducting a planktonic lifestyle. In order to test our hypothesis, the strain G7 of the common soil dweller *Pseudomonas putida*, with the ability to metabolize PAHs (Cébron et al., 2008), was grown in the presence and absence of different SPs (i.e., quartz sand, clays and humic acids) using phenanthrene or glucose as the sole C and energy source. A comparison between PAHs and a common C/energy source could serve indeed to identify specific molecular markers indicative of PAHs metabolism (Wick et al., 2003; Keum et al., 2008). Phenanthrene was used as a model PAH compound because it is relatively easy to be degraded and is ubiquitous in contaminated soils. Glucose, a simple carbohydrate that is less adsorbed on SPs (Wu et al., 2014c), was used to discriminate the C-source effect from the SPs one. The characterization of cell metabolic profiles in the presence of different SPs was carried out by using a combination of vibrational spectroscopy techniques such as Fourier Transform Raman and Infrared spectroscopy (FT-Raman and FTIR spectroscopy, respectively) and the spectra processed by chemometric analysis, such as multivariate classification analysis. Vibrational spectroscopy was preferred over other -omics techniques such as proteomics and transcriptomics because it requires minimal sample preparation, allow high spatial resolution measurements and it provides quantitative and qualitative (i.e., structural) information concerning the whole cell metabolic profile (Naumann, 2001; Huang et al., 2007; Wagner et al., 2010, 2014; Teng et al., 2016; Fanesi et al., 2017). Proteomics and transcritomics look indeed at only one cellular pool at time and they may not provide information concerning the final metabolic phenotype of a cell as a consequence of post-translational modifications (Wagner, 2009). The present work is a pilot study allowing to prove the concept that vibrational spectroscopy techniques may bring interesting and contrasting information complementary to other approaches in soil studies. Furthermore, in view of future applications, vibrational spectroscopy coupled to stable isotope probing (i.e., ^13^C-labeled phenanthrene) would allow the *in situ* identification of specific bacterial functions.

## Materials and Methods

### Culture Conditions and Experimental Setup

*Pseudomonas putida* PpG7 (ATCC^®^17485^TM^; provided by Dr. G. J. Zylstra) was selected as experimental organism for this study because already screened for phenanthrene metabolism (Cébron et al., 2008) and because able in the presence of phenanthrene to sustain enough biomass production required to perform the measurements. Stock cultures were maintained in the dark at 24°C in Bushnell Haas (BH, Sigma-Aldrich; Bushnell and Haas, 1941) agar plates containing glucose (4 g⋅L^-1^). Before streaking the cells, the agar plates were sprayed with 500 μL of a solution of phenanthrene (Sigma-Aldrich) in acetone (10 mg⋅mL^-1^) and the solvent was let evaporate in a laminar flow hood (Thomas et al., 2016).

Experiments were performed on liquid batch cultures growing in 250 mL Erlenmeyer flasks filled with 100 mL of BH mineral medium using either phenanthrene or glucose at a final dosage of 1 mg⋅mL^-1^.

Coarse quartz sand (3–5 mm diameter), Na-montmorillonite (Wyoming) SWy-2 (Source Clay Minerals repository, Chantilly, VA, United States), Na-nontronite NAu-1 (Source Clay Minerals repository, Purdue, IN, United States) and humic acids (53680, Sigma-Aldrich) were selected as representative soil particles for the experiments. The soil particles were used at a concentration of 300, 10, and 0.1 g⋅L^-1^ for sand, clays (montmorillonite and nontronite) and humic acids, respectively. Sand and clays were deposited at the bottom of the flasks in dried forms, whereas humic acids were supplied dissolved in 0.1M NaOH. The flasks were then autoclaved, and the phenanthrene-acetone solution was spread over the whole surface occupied by the particles. In the presence of humic acids, phenanthrene was first deposited at the bottom of the flask, let the acetone evaporate and then the humic acid solution was added. After the complete evaporation of the solvent (previously tested), BH mineral medium was added and the flasks were allowed to equilibrate for at least 4 h [a time pre-established to lead to a complete adsorption of PAHs to mineral particles; (Müller et al., 2007)] on a rotatory shaker (90 rpm) in the dark at 24°C. At this point, an inoculum from an overnight grown agar culture was prepared to obtain a final colony forming unit (CFU) number of 1–2.10^3^ CFU⋅mL^-1^ (determined by plate counting on BH medium supplemented with glucose).

### Growth Rates (μ) and C-dynamics

Growth curves were determined over a period of ∼100 h, on cultures grown under the same conditions described above. Cells of *P. putida* were plate counted after vigorously vortexing the samples for 5–10 min to allow cells that eventually adhered to the SPs to detach. Growth rates (*μ*) were estimated as the slope of a linear least square regression of the natural logarithm of CFU increase against time. Control cultures inoculated without C-source or without cells were also run to exclude possible contamination and artifacts. Each day, samples (1–2 mL of culture) were harvested at different time of the day to perform Raman and FTIR measurements (see below).

On a different set of cultures grown under the same experimental conditions described above, the mineralization efficiency of the cells (measured as CO_2_ production over a time period of ∼100 h) was determined in sealed bottles (150 mL) filled with 10 mL of cell culture. The measurements were performed as described elsewhere (Cébron et al., 2013). Briefly, 3 mL of the bottle atmosphere was sampled with a plastic syringe through a rubber stopper and CO_2_ quantified by and infrared gas analyzer (Binos 1004; Rosemount). After the measurements, the bottles were opened and brought to equilibrium with the external atmosphere in a laminar flow hood. Mineralization curves (CO_2_ emission vs. time) were fitted using the “grofit” package (Kahm et al., 2010) present in the R software (R Development Core Team, 2010) to determine quantitative parameters of CO_2_ production such as the maximum mineralization (max value at plateau) and mineralization rate (the initial slope of the CO_2_ evolution vs. time curve).

To better understand the dynamics of the C-source in the presence of the different SPs, the soluble fractions of phenanthrene and glucose (i.e., concentration in solution) were quantified at the beginning (before cell inoculation) and at the end of the experiments (after ∼100 h). Due to the low amount of phenanthrene in solution the whole volume of growth medium in a flask must be utilized for its quantification. Therefore, another set of experiments was run to determine the soluble fraction of phenanthrene and glucose. For phenanthrene quantification, three cultures for each time point were prepared and inoculated. For each time point, the whole cultures were centrifuged at 16,000 ×*g* for 5 min to completely remove SPs and cells in suspension. Soluble phenanthrene was then extracted twice in dichloromethane (DCM), in 8 and 5 ml respectively. The DCM containing phenanthrene was then evaporated in dark glass tubes under a N_2_ flux and substituted with acetonitrile for phenanthrene quantification using HPLC (see Thomas et al., 2016). For glucose quantification, 1mL of culture was centrifuged (as above) and filtered (0.22 μm pore size). Glucose was finally quantified using the GOD-PAP kit (Bioloabo, Maizy, France) and a Safas MP96 spectrophotometer (Safas, Monaco).

### FT-Raman Micro-Spectroscopy and Diffuse Reflectance FTIR-Spectroscopy: Sample Preparation and Spectra Acquisition

The metabolic profile of *P. putida* was characterized by a combination of vibrational spectroscopy techniques. Samples were harvested each day (multiple times during the day, typically every 2 h; for a total of 4–5 samples per day) over the whole growth period (see above) to exclude compositional differences related to the growth phase. Cells (1–2 ml of culture) were harvested by centrifugation at 10,000 × *g* for 4 min and washed twice in sterile MilliQ water to remove residual salts and cell debris. The cell pellet was then resuspended in 5–10 μL of MilliQ water and 0.5 μl of cell suspension was deposited on a gold coated microscopy slide and let dry at room temperature. Afterward, the cell deposit was resuspended in 0.7 μL of 0.9% (w/v) NaCl and dried again. In the presence of SPs, cells were separated from the particles by vigorously vortexing the samples for 5–10 min and by further sonicating them in an ultrasonic bath for few seconds. In the presence of clay, cells were further separated from the mineral particles (which were found to produce a strong fluorescence background in the Raman spectra) by density gradient using Nycodenz solution (1.3 g⋅mL^-1^; ProteoGenix, Axis-Shield). Macro-aggregates were removed by a short spin centrifugation step. The remaining suspension (1 mL) was vortexed and sonicated as described above and Nycodenz (0.8 mL) was carefully added at the bottom of the eppendorf. The samples were centrifuged at 3,000 ×*g* for 20 min and the cell layer interposed between the aqueous phase and the Nycodenz removed and washed in MilliQ water at least 5 times to eliminate any trace of Nycodenz that could have interfered with cell signals.

In this study the use of a Fourier Transform (FT-) Raman spectrometer operating in the near infrared was preferred to a dispersive Raman device using visible lasers to avoid signal distortions due to native fluorescence of PAHs and of soil components (i.e., the SPs). Raman scattering was acquired using a FT-Raman MultiRam spectrophotometer (Bruker, Ettlingen, Germany), equipped with an excitation line at 1064 nm (Nd:YAG laser) and a liquid nitrogen cooled high sensitivity Ge detector. The spectrometer was coupled to an epifluorescence right microscope (BX51 Olympus) equipped with a 50X magnification IR objective (LCPLN50XIR; WD 4-5mm, NA 0.65, Olympus, Japan), specific for optimal transmission in the near infrared field between 700 and 1300 nm (Transmittance up to 75% in the range 0–2000 cm^-1^). Spectra were acquired in reflection mode in the spectral range 4000–0 cm^-1^ with a resolution of 6 cm^-1^ by recording 1000–2000 scans at 2.2 kHz of slew rate in order to get good signal to noise ratios. Spectra were processed using Blackman-Harris apodization function and 2 levels of zerofilling. The laser power used for the measurements was 500–600 mW corresponding to approximately 200 mW at the sample. Samples were scanned at least at 2 different positions, thanks to an x, y, z motorized stage, to account for sample heterogeneity. Spectra acquisition was controlled with the OPUS 7.5 software (Bruker, Ettlingen, Germany). The resolution of the instrument (∼10–20 μm) does not allow single cell analysis, therefore the spectra reported in this study refer to bulk analysis including both cells that were adhered to the SPs and the planktonic ones.

The same set of samples prepared for the FT-Raman measurements were also scanned by means of FTIR-spectroscopy to obtain complementary information about the metabolic profiles of *P. putida* populations. Diffuse reflectance spectra (DRIFTS) acquisition was carried out with a Bruker Vector 22 (Bruker, Ettlingen, Germany) equipped with a diffuse reflection collection system (Praying Mantis; Harrik, Pleasantville, NY, United States). Spectra were recorded with 64 scans co-added and averaged in the spectral range 4000–400 cm^-1^ with a resolution of 4 cm^-1^. Background spectra were recorded at the edges of the cell deposit with the same instrumental settings. The spectrometer was controlled with the OPUS software version 4.5 (Bruker, Ettlingen, Germany).

Band assignment for the Raman and IR spectra was based on literature references (Naumann, 2001; Movasaghi et al., 2007, 2008; Huang et al., 2010) and on reference spectra of pure macromolecules.

### Spectra Pre-processing and Multivariate Modeling

All spectra were exported from the OPUS software for further pre-processing and multivariate modeling steps computed in the R environment (version 3.4.3; R Development Core Team, 2010). A first visual screening, aimed at eliminating the spectra with a low signal to noise ratio, resulted in a total of 346 for the FT-Raman dataset and 465 for the FTIR one. Base line corrections of Raman spectra were performed with the package “baseline” (Liland et al., 2015) using a 2nd derivative constrained weighted regression, cut in the spectral ranges 3019-2819 and 1780-400 cm^-1^ and normalized (in order to compare peak intensities and remove problems related to different sample thickness) using the standard normal variate function (SNV, Barnes et al., 1989). FTIR-spectra were converted to 2nd derivatives by the Savitzky-Golay algorithm (Savitzky and Golay, 1964) using a quadratic polynomial function with nine smoothing points. Prior to calculations, spectra were cut in the spectral ranges 3019–2819 and 1780–1200 cm^-1^ because of the strong clay bands overlapping to the cellular components in the lower frequency range of the IR spectrum. However, other two bands corresponding to the spectral ranges, 1091–1076 and 973–958 cm^-1^ were further selected because not overlapping with clay signals. Finally, the spectra were normalized using the SNV function.

The Partial Least Square (Wold et al., 2001) Discriminant Analysis (PLS-DA; Barker and Rayens, 2003) was used to unravel consistent patterns in the spectral datasets and to extract information regarding the macromolecules involved in the classification. The PLS-DA is a chemometric analysis particularly useful when it comes to handle metabolic datasets with highly collinear variables (Fonville et al., 2010). The PLS-DA was implemented in the R software using the ‘pls’ package developed by Mevik et al. (2016). To test our hypothesis, two models for each spectral dataset were developed. The first discriminated each experimental treatment (control and the 4 SPs and the 2 respective C/energy source; 10 classes) and the second discriminated the C/energy sources (i.e., glucose and phenanthrene; 2 classes). The models were calibrated by matching each set of predictors (X-variables; i.e., FT-Raman and FTIR spectra) with the corresponding discriminating classes (Y-variables). The matrices (i.e., Y variables) were created assigning integer values of 0 and +1 (dummy variables) to the experimental treatments (SPs + C/energy source) and C/energy source (glucose and phenanthrene). In turn, to each class is assigned the values +1 and to all the others 0. In this way, a calibration can be built around each classification matrix. To quantify the accuracy of the classification models, the sensitivity (the ability of identifying true positives) and the specificity (the ability of identifying true negatives) were estimated as described in Sackett et al. (2013), using the “caret” package (Kuhn, 2017) present in the R software. Spectra with predicted *Y*-values ≤ 0.5 were classified as 0, whereas predicted *Y*-values ≥ 0.5 were classified as 1. The algorithm used for the PLS-DA was the canonical powered partial least square (Indahl et al., 2009). The prediction abilities of the models were inferred from the root mean squared error of calibration and prediction (RMSEC and RMSEP, respectively), the latter calculated from the leave-one-out cross-validation (LOOCV), and the coefficient of determination (*R*^2^). The *R*^2^ and the RMSEP obtained from the cross validation were used to determine the number of principal components to use for each model (Wold et al., 2001). Class similarities and dissimilarities were individualized by plotting the model’s scores. Important changes in macromolecules and functional groups in response to SPs and C/energy source were identified by extracting and analyzing the loadings of each model. The loadings indicate the variables, i.e., the spectral features (corresponding to specific compounds or functional groups), mainly driving the discriminant model.

### Statistical Analysis

All data are reported as mean and standard deviations of three independent biological replicates. Statistically significant differences between mean values of the measured variables were assessed by a two-way analysis of variance (ANOVA), considering the SPs and the C-source as factors, followed by the Bonferroni *post hoc* test. The level of significance was always set to α = 0.05.

## Results

### Growth Rate (μ)

Growth rate of *P. putida* was affected both by the C-source and by SPs. In general, cells presented lower *μ* when growing on phenanthrene in comparison to glucose (Table 1). With glucose, all SPs but montmorillonite sustained higher *μ* respect to the cultures grown in the absence of SPs. The same trend was observed when the cells were consuming phenanthrene, however, in the presence of montmorillonite the cells exhibited comparable *μ* with respect to the cells grown in the absence of SPs.

**Table 1:** Growth rate (μ), maximal mineralization and final C-source concentrations in the cultures of *P. putida* grown in the absence and in the presence of SPs (sand, clays, and humic acids) and using glucose or phenanthrene as the sole source of C and energy.

	μ (h^-1^)	Maximal mineralization (μg C-CO_2⋅_ml^-1^)	C-source_final_ (μg⋅ml^-1^)
**Glucose**			
No SPs	0.46^a^ (0.00)	280^a^ (1.8)	52.43^a^ (12.97)
Sand	0.58^b^ (0.05)	268.7^a^ (2.1)	14.98^a^ (6.48)
Montmorillonite	0.18^c^ (0.03)	270.5^a^ (9.9)	18.72^a^ (17.16)
Nontronite	0.65^d^ (0.00)	219^b^ (14.5)	48.68^a^ (84.33)
Humic acids	0.63^e^ (0.03)	228.5^c^ (24.5)	89.88^a^ (47.67)
**Phenanthrene**			
No SPs	0.31^a^ (0.00)	106.5^a^ (13.6)	0.12^a^ (0.03)
Sand	0.43^b^ (0.01)	99.5^a^ (9.3)	0.10^a^ (0.01)
Montmorillonite	0.31^a^ (0.01)	112.3^a^ (12.1)	0.41^a^ (0.20)
Nontronite	0.59^c^ (0.00)	51.7^b^ (1.3)	1.18^b^ (0.28)
Humic acids	0.45^d^ (0.00)	39.4^c^ (6.6)	0.95^c^ (0.58)


*Results are reported as mean and standard deviations (in brackets), *n* = 3. Different letters represent statistically significant means (two-way ANOVA, *p* < 0.05) of the parameters in the presence of the SPs, with respect to those measured in the absence of SPs (i.e., No SPs).*

### C-source Soluble Fraction and Mineralization

**FIGURE 1:**
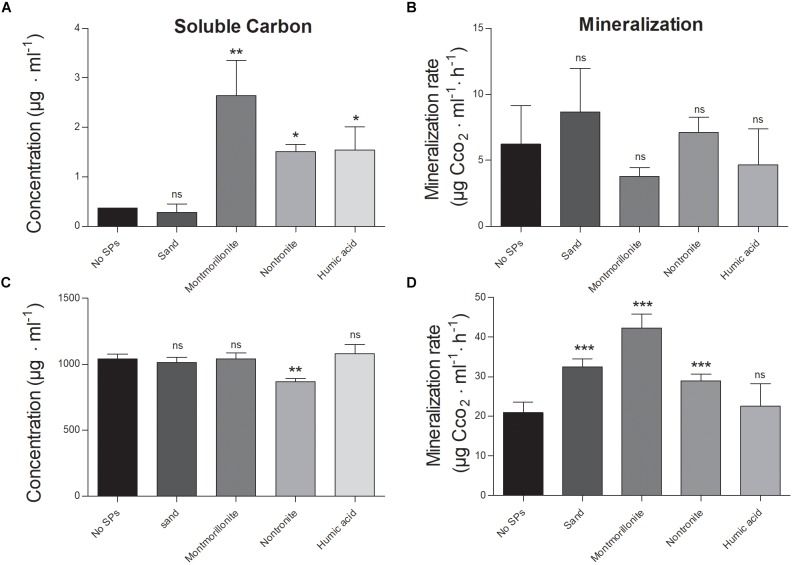
Soluble fractions of the C-sources and mineralization rate of *P. putida* grown in the absence (No SPs) and presence of SPs and metabolizing glucose or phenanthrene as the sole C and energy source. **(A,B)** Depict the soluble carbon and the mineralization rate, in the cultures grown in the presence of phenanthrene. **(C,D)** Depict the soluble carbon and the mineralization rate, in the cells grown in the presence of glucose. Vertical bars represent standard deviations (*n* = 3), statistically significant differences with respect to the cultures containing no SPs (black bars) are also reported (ns, non significant, ^∗^*p* < 0.05, ^∗∗^*p* < 0.01, ^∗∗∗^*p* < 0.001).

In all conditions, the soluble phenanthrene fraction was approximately 0.1–0.2% compared to that of glucose (Figures 1A,C). Montmorillonite, nontronite and humic acids increased the amount of phenanthrene in solution (likely as colloids), with respect to the cultures grown in the absence of SPs, or in the presence of sand (Figure 1A). Interestingly, the soluble fraction of phenanthrene was comparable in the absence of SPs and in the presence of sand, whereas with montmorillonite it was the highest (5 times higher than in the absence of SPs), followed by nontronite and humic acids that supported a similar concentration of phenanthrene in solution (3 times higher than in the absence of SPs) (Figure 1A). On the other hand, glucose was dissolved completely at all conditions but in the presence of nontronite the soluble fraction was 18% lower (Figure 1C).

At the end of the experiment, only 1–10% of the initial glucose was left available for the cells in all the conditions and no effect of the SPs on the total consumption of glucose could be detected (which ranged between 90 and 99% of the initial amount) (Table 1). While montmorillonite increased the concentration of soluble phenanthrene at the beginning of the experiment, the concentration of soluble phenanthrene was the lowest in this condition at the end of the experiment, and no difference between all the other conditions was evident (Table 1).

The mineralization efficiency of *P. putida* was assessed by means of CO_2_ assays (i.e., CO_2_ evolution over time). The maximal CO_2_ production and the initial slope of the mineralization curves were 3–4 times lower when the cells were grown on phenanthrene, with respect to glucose (Figures 1B,D and Table 1). Regardless of the C-source, SPs induced only minor changes of the maximal mineralization attained by the cells (Table 1). Nontronite and humic acids sustained the lowest maximal mineralization level, whereas at all other conditions the cells presented comparable values (Table 1). When growing on phenanthrene, the cells did not show any change of the mineralization rate induced by the presence of the SPs (Figure 1B). On the other hand, the mineralization rate was strongly affected by SPs when glucose was the C-source (Figure 1D). Montmorillonite particles sustained the fastest mineralization, whereas the mineralization rate decreased with nontronite and sand particles (Figure 1D). In the absence of SPs and in the presence of humic acids, the cells exhibited instead the lowest mineralization rates.

### Metabolic Profiling by FT-Raman Spectroscopy and PLS-DA

**FIGURE 2:**
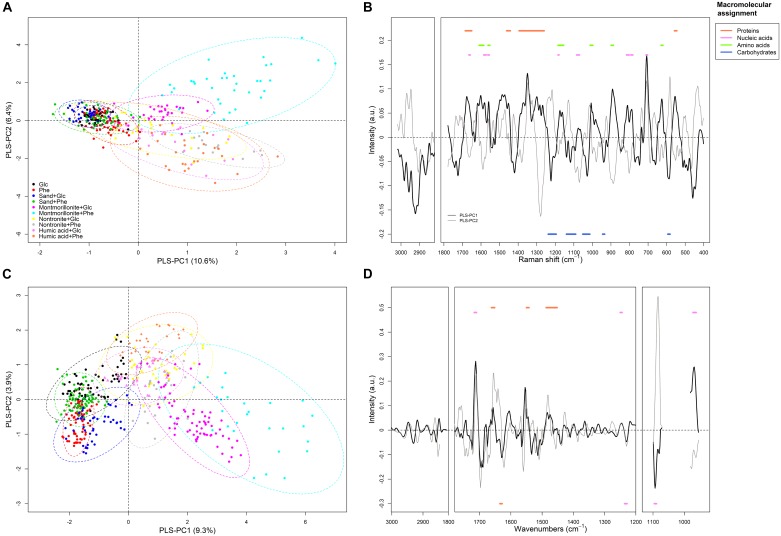
Partial Least Square Discriminant analysis (PLS-DA). The PLS-DA was calibrated for the discrimination of each experimental treatment [i.e., ±SPs and glucose (Glc) or phenanthrene (Phe)] based on FT-Raman and FTIR-spectra of *P. putida*. The panels **(A,C)** report the scores of the FT-Raman and FTIR model, respectively. The panel **(B,D)** represent the loadings of the FT-Raman and FTIR model, respectively. For the FT-Raman model, a positive loading corresponds to a higher signal intensity (for a compound) in all the samples presenting positive scores. For the FTIR model, since the spectra were converted to 2nd derivatives a positive loading corresponds to a lower signal intensity (for a compound) in all the samples presenting positive scores. Dots are spectra of *P. putida* samples (i.e., including planktonic and adhered cells) acquired at each condition over the whole growth curve. Dashed ellipses in the score plots represent the 95% confidence ellipses for each class. Horizontal bold lines in the loading plots represent the macromolecular assignment of the peaks. The sign (+ and –) of the bold lines reflect that of the corresponding loading of the PLS-PC1.

The average FT-Raman spectra for each experimental condition are reported as Supplementary information (Supplementary Figure S1). Differences all over the spectral ranges could be detected among all conditions, however, for a precise identification of patterns, the use of chemometric analysis was necessary. The first PLS-DA model was calibrated using the first eight principal components (PLS-PC) for the classification of each of the growing conditions (i.e., the different type of SPs and C-source) on the base of *P. putida* FT-Raman spectra. From a visual inspection of the scores plot (where each sample is represented in a bi-dimensional space), it emerged that differences in the metabolic profiles of *P. putida* could be used to discriminate among the different growth conditions, with the SPs being the main factor influencing the discriminant analysis (Figure 2A). The first eight components explained 37% of the overall variance present in the spectral dataset (i.e., the X matrix) (Supplementary Table S1). The best discrimination, and therefore the greatest differences in the metabolic profiles of the cells, occurred for the cells grown in the presence of montmorillonite, nontronite and humic acids (Table 2 and Figure 2A). The combination of these SPs with phenanthrene, as a C/energy source, resulted in the highest variance explained (77, 65, and 71%, respectively) (Table 2). The higher degree of macromolecular changes was also confirmed by the higher sensitivity (i.e., the ability of the model to discriminate those classes of cells from the rest of the treatments) for montmorillonite, nontronite and humic acids in the presence of phenanthrene being 69, 43, and 69% respectively (Table 2). For these classes, the model also presented the higher *R*^2^_C_ (0.77, 0.64, and 0.70, respectively) and the lower RMSEP (0.17, 0.15, and 0.17, respectively), indicating good predictive abilities.

**Table 2:** Diagnostic parameters of the PLS-DA models based on FT-Raman and FTIR spectroscopy.

	Glc	Phe	S + Glc	S + Phe	M + Glc	M + Phe	N + Glc	N + Phe	HA + Glc	HA + Phe
**FT-Raman**										
Sensitivity	0.01	0	0	0.02	0.29	0.69	0	0.43	0	0.69
Specificity	0.99	0.99	1	0.99	0.97	0.99	0.99	0.99	0.99	0.98
*R^2^*_C_	0.25	0.18	0.17	0.27	0.55	0.77	0.43	0.64	0.37	0.70
RMSC	0.31	0.33	0.25	0.29	0.19	0.14	0.19	0.12	0.18	0.14
*R^2^*_P_	0.14	0.07	0.04	0.11	0.20	0.68	0.16	0.43	0.009	0.56
RMSEP	0.34	0.36	0.27	0.32	0.25	0.17	0.23	0.15	0.22	0.17
% variance	25.86	18.47	17.19	27.98	55.19	77.03	43.27	64.89	37.65	70.58
**FTIR**										
Sensitivity	0.70	0.01	0.17	0.34	0.80	0.75	0.29	0.76	0	0
Specificity	0.98	0.99	1	0.98	0.97	1	1	1	1	1
*R^2^*	0.51	0.28	0.37	0.36	0.59	0.67	0.45	0.65	0.21	0.32
RMSC	0.25	0.28	0.23	0.28	0.22	0.13	0.19	0.13	0.19	0.19
*R^2^*_P_	0.46	0.21	0.24	0.26	0.44	0.55	0.28	0.43	0.10	0.25
RMSEP	0.25	0.29	0.24	0.29	0.23	0.14	0.20	0.14	0.20	0.20
% variance	51.08	28.84	37.48	36.83	59.64	67.66	45.80	65.82	21.71	32.22


*The models were calibrated to discriminate for each experimental condition (± SPs and glucose or phenanthrene), for a total of 10 classes (Glc, glucose; Phe, phenanthrene; S, sand; M, Montmorillonite; N, Nontronite; HA, humic acids). The number of principal components was 8 for both models. RMSC, root mean squared error of calibration; RMSEP, root mean squared error of prediction; *R^2^_C_* coefficient of determination for the calibration; *R^2^_P_* coefficient of determination for predicted values; Sensitivity, ability to discriminate true positives; Specificity, ability to discriminate true negatives; % variance, variance in Y explained by the model.*

The spectra corresponding to cell grown on montmorillonite, nontronite and humic acids grouped at positive scores along the PLS-principal component one (PLS-PC1; 10.6%), whereas the cells grown in the absence of SPs and in the presence of sand clustered at negative ones (Figure 2A). On the other hand, along the PLS-PC2 (6.4%) a discrimination of the different SPs could be observed (Figure 2A). Spectra corresponding to cells grown in the presence of montmorillonite were present at positive scores and the cells grown on nontronite and humic acids had negative ones (Figure 2A).

The loadings, together with the patterns observed in the scores plot, indicate which spectral bands (i.e., metabolic components) are responsible for the observed clustering patterns. The complete band assignment is reported in Supplementary Table S2. For the PLS-PC1, a set of important loadings corresponding to carbohydrates spectral window (C-O and C-C stretching; 1200–1000 cm^-1^) and carboxylic residual groups (symmetric vibrations of carboxylate groups COO^-^ near 1419 cm^-1^) had negative sign, indicating that the cells growing in the absence of SPs or in the presence of sand (also found at negative scores) exhibited higher peak intensities for this macromolecular pool and residual group, compared to cells grown in the presence of clays and humic acids (Supplementary Table S2 and Figure 2B). Positive loadings corresponded to the C = O stretch of the Amide I, present at 1675 cm^-1^, the Amide III (1265 cm^-1^), and the CH_2_ bending of proteins (1452 cm^-1^). Characteristic vibration frequencies of amino acids such as phenylalanine (1600, 1174/1162, 1006, and 626 cm^-1^) tyrosine and tryptophan (1600, 1558, 1174/1162, 891 cm^-1^), ring breathing modes of purine bases (Adenine and Guanine rings at 1581, 788, and 707 cm^-1^) and the symmetric PO_2_ stretching of DNA (1076 cm^-1^) presented positive sign. They were therefore more important in the cells grown in the presence of clays and humic acids (found at positive scores).

For the PLS-PC2, negative loadings corresponding to proteins (1662, 1278 cm^-1^), amino acids (1602, 1000, 889, and 802 cm^-1^) and nucleic acids (1591, 1560, and 1016, 639, 619 cm^-1^) indicated more intense bands for these components in the cells growing in the presence of nontronite and humic acids. With montmorillonite instead, the cells presented positive loadings for most of the carbohydrates bands (1000–1200 cm^-1^) and carboxylic residual groups (COO^-^ at 1419 cm^-1^).

**FIGURE 3:**
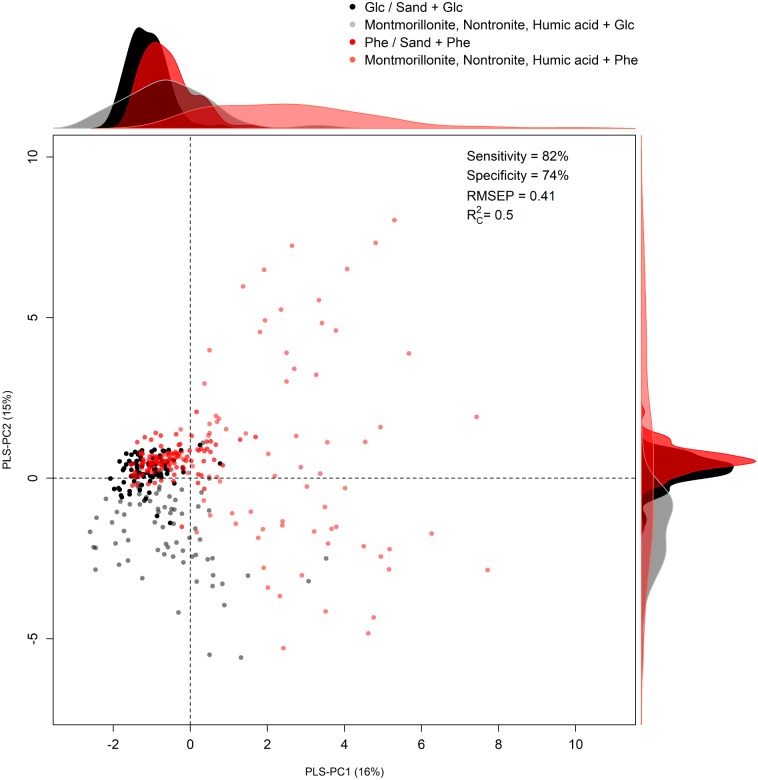
Partial Least Square Discriminant analysis (PLS-DA). Scores plot of the model calibrated to discriminate the C-source [phenanthrene (Phe) and glucose (Glc)] based on FT-Raman spectra. Density distributions along the axes were drawn to help patterns visualization. The summary statistics (sensitivity, specificity, RMSEP, and *R*^2^_C_) of the discriminant model is also reported.

The second model was calibrated (using four PLS-PC) to discriminate for the C/energy source (glucose and phenanthrene). Even in this case, patterns in the two classes of cells could be discriminated based on their metabolic profiles (Figure 3). The first four components explained 38% of the total spectral variance, whereas the explained variance for the C/energy source was 50% and the RMSEP was 0.41 (Supplementary Table S3). Furthermore, the sensitivity and specificity were high (82 and 74% respectively), meaning that the differences in macromolecule composition were strong enough to allow for accurate classification (Supplementary Table S3). Analyzing the scores plot, an interesting pattern in the metabolic profiles could be observed. When *P. putida* was grown in the absence of SPs, or in the presence of sand, the cells presented comparable metabolic profiles (i.e., overlapping scores) regardless of the C-source metabolized (i.e., glucose or phenanthrene) (Figure 3). On the other hand, when montmorillonite, nontronite, and humic acids were present in the medium, a greater discrimination among the cells grown in the presence of glucose or phenanthrene was evident (Figure 3). For instance, along the PLS-PC1 (16% of total variance) the cells grown on glucose and in the presence of SPs were present at negative scores and the cells grown on phenanthrene and SPs had positive ones. In between these two groups clustered the cells grown in the absence of SPs or in the presence of sand (Figure 3). The PLS-PC1 positive loadings revealed that when using phenanthrene, the cells presented greater signals for components such as proteins (1675, 1282 cm^-1^), and nucleic acids (794 and 707 cm^-1^) (Supplementary Figure S2). On the other hand, when growing on glucose the cells had higher intensities for carbohydrates (1200–1000 cm^-1^) (Supplementary Figure S2). The variance along the PLS-PC2 (15%) was mainly due to phenylalanine (1589 cm^-1^), the Amide III (1286 cm^-1^), which presented strong negative loadings, and carbohydrates (582 cm^-1^) with positive one (Supplementary Figure S2).

### Metabolic Profiling by FTIR Spectroscopy and PLS-DA

The average FTIR spectra for each experimental condition are reported as Supplementary Figure S3. Even in this case, the first model was calibrated to discriminate the different growth conditions (i.e., SPs and C/energy source). Similarly to the respective FT-Raman model, the first eight components explained 38% of the total spectral variance present in the dataset (Supplementary Table S1). The highest variance explained by the spectra was reached with glucose and in the absence of SPs, in the presence of montmorillonite combined with phenanthrene or glucose and nontronite with phenanthrene, explaining 51, 68, 60, and 66% of the variance respectively (Table 2). For these set of conditions, the model presented also the highest sensitivity, specificity and the best predictive abilities (Table 2), reflecting the greatest changes in the macromolecular composition of the cells. In the scores plot, the most prominent discrimination between the conditions occurred along the PLS-PC1 (9%). The cells grown in the absence of SPs and in the presence of sand presented comparable metabolic profiles and were grouped at negative scores (Figure 2C). Moving to positive scores, the cells grown with montmorillonite, nontronite, and humic acids in the medium could be found. No clear separation in the presence of these last SPs could be detected and the spectra presented overlapping scores (Figure 2C). No clear trend along the PLS-PC2 was evident.

Since the FTIR-spectra were transformed to 2nd derivatives, positive loadings correspond to a decrease in intensity of the specific compounds/residual groups for all the spectra presenting positive scores. The opposite holds true for samples with negative scores. The loadings corresponding to C-H bonds vibrations (3000–2820 cm^-1^) presented only minor contribution to the discriminant model (Figure 2C). Important positive loadings showed that the cells growing in the absence of SPs or in the presence of sand presented higher signals for the C = O stretching of the carbonyl groups contained in lipids (1743 cm^-1^) and in nucleic acids (1712 cm^-1^). Protein signals corresponding to Amide I α-helix (C = O stretching; 1656 cm^-1^) and Amide II (N-H bending; 1544 cm^-1^) together with backbone features of nucleic acids (C-C stretching; 968 cm^-1^) and the asymmetric PO_2_^-^ stretching (1245 cm^-1^) also presented positive loadings (Figure 2D). Negative loadings for the model corresponded to ß-sheets protein secondary structures (1675 and 1629 cm^-1^), and to the symmetric stretching of PO_2_^-^ groups present in phosphorylated molecules (1230 and 1093 cm^-1^) (Figure 2D).

The second model was calibrated to discriminate for the C-source. The first eight components captured 87% of the total spectral variance (Supplementary Table S3). The REMSP was 0.28, the *R*^2^_C_ was 0.71 and sensitivity and specificity were 92 and 94%, respectively. Although the PLS-PC1 and PLS-PC2 already explained 50% of the total variance in the spectral dataset, no clear distinction between the cells grown on glucose and on phenanthrene could be observed (Supplementary Figure S4). The only separation, but rather weak, was evident along the PC2 (14% of variance) between the cells grown in the absence of SPs or in the presence of sand and the one grown on clays and humic acids. However, no separation occurred on the basis of the C-source, as shown by the overlapping scores (Supplementary Figure S4). The clustering pattern in the scores plot was mainly driven by changes in the secondary structures of proteins (1658 and 1637 cm^-1^) and carbonyl groups of nucleic acids (1708 cm^-1^), as suggested by the loadings of the PLS-PC1 and PLS-PC2 (Supplementary Figure S5).

## Discussion

Soil is a complex matrix represented by a highly heterogeneous association of mineral and organic components. These constituents not only influence the physico-chemical conditions that microorganisms are subjected to, but they also affect the availability and the spatial distribution of organic pollutants, such as PAHs. Understanding how bacteria metabolically react to the presence of different soil components during the mineralization of PAHs is therefore of paramount importance to understand pollutants degradation in nature and to improve remediation strategies of polluted sites. Up to date, little information in this regard is available (Ortega-Calvo and Saiz-Jimenez, 1998; Uyttebroek et al., 2006; Lerch et al., 2017). In this study, for the first time we aimed at studying how different type of soil particles (SPs) influence the metabolic profile of bacteria grown in the presence of phenanthrene.

### Soil Particles Differently Affect the C-source in Solution and Cell Mineralization Efficiencies According to the Nature of the C-source (Phenanthrene vs. Glucose)

Our results show that in the presence of glucose, the SPs modified the mineralization efficiencies of the cells, though no relevant difference in the soluble fraction of the C-source could be detected (Figure 1 and Table 1). The higher metabolic performance (i.e., CO_2_ production) in the presence of montmorillonite is consistent with the results of Wu et al. (2014c), who ascribed the changes in metabolic activity (measured by microcalorimetry) to the SPs presence itself, rather than to changes in C solubility. In the presence of phenanthrene, the SPs specifically altered the amount of phenanthrene in solution. Sand, which presents the lowest specific surface area among the SPs tested (Wilcke et al., 1996; Amellal et al., 2001; Uyttebroek et al., 2006; Müller et al., 2007), did not alter the phenanthrene concentration in solution (Louvel et al., 2011). On the other hand, montmorillonite, nontronite and humic acids increased the amount of readily available phenanthrene in solution, maybe as colloids. Although in contrast with the finding reported by Ortega-Calvo and Saiz-Jimenez (1998), we have to point out that the presence of colloidal fractions of SPs might have acted as surfactants and increased the apparent solubility of phenanthrene in the aqueous phase of the cultures as described by Kanti Sen and Khilar (2006). Besides the amount of phenanthrene that each SP is able to adsorb, other physical parameters, such as the contact surface area of a particle and the aqueous phase, may also affect the phenanthrene flux from the surface of the particle into the surrounding medium as a function of the substrate uptake by the cells (Wick et al., 2001; Tecon et al., 2006). Despite of this, the mineralization parameters (e.g., maximum mineralization and mineralization rate) were similar in all treatments, meaning that the soluble fraction was only one of the exploitable phenanthrene-pools present in the cultures (Ortega-Calvo and Saiz-Jimenez, 1998; Amellal et al., 2001), and that the cells presented efficient mechanisms to increase their accessibility to phenanthrene to efficiently biodegrade it.

From these results, it is evident that SPs have opposite effects on C accessibility and cell metabolic activity as a function of the C-source present in the growth environment: with glucose, SPs had an action on cell metabolic activity, whereas in the presence of phenanthrene they appeared to modify more the access to the C-source. Although this finding is an evident consequence of the contrasting physico-chemical properties of glucose and phenanthrene, less obvious are the metabolic adjustments of the cells facing these two opposite scenarios.

### Soil Particles Specifically Alter the Metabolic Profile of *P. putida*

The metabolic profile of *P. putida* was characterized by vibrational spectroscopy. FT-Raman and FTIR-spectra carry complementary information concerning bacteria macromolecular composition. When run in parallel, these techniques can draw a precise picture of cellular biochemical changes, which can be extracted by advanced statistical analysis (i.e., chemometrics). The first PLS-DA model was calibrated considering each single treatment (SPs + C-source) as a different class.

Although FT-Raman-spectra, with respect to FTIR-spectra, present a lower degree of overlapping bands and more detailed spectral information about amino acids, nucleic acids bases and aromatic compounds (Neugebauer et al., 2007), overall, the models classifying for each condition (SPs + C-source) were quite similar (Figure 2). The PLS-DA revealed that most of the macromolecular changes are related to cells grown in the presence of montmorillonite, nontronite and humic acids with no significant effect due to the presence of sand (Figures 2A,C). Interestingly, from the scores plot it also emerged that the cells could be well discriminated in the presence of different SPs (Figures 2A,C), confirming our hypothesis that SPs specifically alter cell metabolic profiles. Furthermore, as indicated by the diagnostic tools of the PLS-DA model (such as sensitivity, *R*^2^ and RMSEP; Sackett et al., 2013), the strongest macromolecular changes were induced when montmorillonite was present in the culture medium, regardless of the C-source, or when clays and humic acids were added to the cultures grown with phenanthrene (Table 2), suggesting an interaction of SPs and C-source in defining the metabolic profile (see below) with different underlying mechanisms as explained below.

To understand which cellular compounds (or functional groups) drove the compositional patterns at the different conditions, we analyzed the loadings of the discriminant models (Wold et al., 2001; Sackett et al., 2013). The biochemical differences distinguishing the cells grown in the absence of SPs or in the presence of sand, from the ones grown on clays and humic acids, concerned mainly changes of the protein, nucleic acid and carbohydrate pools, with the latter one being negatively correlated to the others (Figures 2B,D). The identified spectral ranges (and therefore the corresponding compounds), especially the one indicated by FT-Raman spectroscopy, have already been described to differentiate between planktonic and biofilm bacteria phenotypes (Andrews et al., 2010), suggesting a possible preference for a sessile lifestyle in the presence of clays and humic acids. However, since in our experimental design the planktonic and attached fraction of bacteria were not separated but considered as a whole, we are not able at the moment to disentangle the direct (adhesion) from indirect effect (C-availability) of the presence of SPs on cell metabolic profile.

The presence of clays and humic acids induced a shift toward β-sheet secondary structures as indicated by the FT-Raman-based loading at 1675 cm^-1^ (Figure 2B) (Rygula et al., 2013). Similar qualitative changes were also detected by FTIR spectroscopy. In the absence of SPs and in the presence of glucose, cells exhibited a prominent Amide I peak at 1656 cm^-1^ in the 2nd derivative spectra (Supplementary Figure S3), indicative of proteins dominated by α-helic secondary structures. In all other conditions, the loadings indicated a shift toward proteins with more β-sheets secondary structures (e.g., 1630 cm^-1^, parallel β-sheets) (Figure 2D) (Barth, 2007). Previous studies demonstrated that the adhesion of bacteria to mineral surfaces can be mediated by protein bridging favored by the presence of α-helic structures (Parikh and Chorover, 2006; Wu et al., 2014a,b). Therefore, in this case the opposite trend suggests that proteins pool modifications were not related to particle adhesion. On the other hand, a higher fraction of β-sheet secondary structure is in line with a reorganization toward a more functional protein pool, indeed β-sheet secondary structures are mostly characteristic of membrane bound enzymes and transporters (Rygula et al., 2013). Such a shift could have been aimed at optimizing the access and acquisition to the C-source, especially in the presence of phenanthrene.

The nucleic acid pool was also affected by the presence of clays and humic acids and the changes were detected both by FT-Raman and FTIR spectroscopy. The loadings of the FT-Raman model indicated a positive correlation between nucleic acids and proteins (Figure 2B). This correlation reflects the biological link between the synthesis of new proteins (see above) and DNA translation that can be easily monitored by means of vibrational spectroscopy (Neugebauer et al., 2007). Whereas the Raman spectrum of nucleic acids contains mainly information about nucleobases, FTIR spectra are informative for backbone vibrations of DNA and RNA. The loadings of the FTIR-based model pointed at five interesting bands (1712, 1245, 1230, 1093, and 968 cm^-1^) (Figure 2D). The pattern of these bands suggests possible changes of DNA molecules conformation (Whelan et al., 2011, 2014) when clays or humic acids were present in the cultures. Whelan et al. (2011, 2014) described in detail the diagnostic bands indicative of DNA conformational shift and how they can be detected by FTIR-spectroscopy. In prokaryotes, DNA conformational changes are responsible for a greater stability upon UV, chemicals and desiccation exposition or as a normal consequence of biological processes such as gene transcription and DNA-protein interactions (Mohr et al., 1991; Whelan et al., 2011, 2014). This conformational changes occurred independently from the C/energy source and they were therefore likely triggered by the presence of SPs (see Supplementary Figure S3).

Carbohydrates were found to be negatively correlated with proteins and nucleic acids (see the FT-Raman loadings). An inspection of the 1200–950 cm^-1^ region of the FTIR spectra suggested that the carbohydrates bands were not originating from storage compounds such as glycogen, but were rather belonging to cell wall structures (in the case of montmorillonite and nontronite a subtraction of pure clay spectra was performed; data not shown) (Jiang et al., 2004). A similar capsular structure seems to surround both planktonic and biofilm entangled cells of *Pseudomonas putida* (Kachlany et al., 2001). In agreement with our findings a spectroscopic analysis reported a lower amount of carbohydrates in *Pseudomonas* sp. NCIMB 2021 for the biofilm lifestyle (Beech et al., 1999) indicating ongoing modifications of surface carbohydrates in the presence of clays and humic acids.

Besides the main metabolic differences found to distinguish the cells grown in the absence of SPs or in the presence of sand and the one facing clays and humic acids, the PLS-DA revealed that the metabolic profile of *P. putida* was specifically affected by the SPs used in this study. For instance, the PLS-PC2 discriminated among montmorillnite, nontronite and humic acids. Wu et al. (2014c) recently demonstrated that the metabolic activity of *P. putida* was peculiarly modulated by different soil colloids and minerals. In accordance to this study, we found that the effect of SPs is also reflected in a specific reorganization of their metabolic profile.

### The Effect of the C-source (Phenanthrene vs. Glucose) on *P. putida* Metabolic Profile Is Evident Only in the Presence of Clays and Humic Acids

From the first PLS-DA model statistics, differences in the metabolic profile of *P. putida* related to the C-source were identified. To better resolve these variations, a second set of models (one based on the FT-Raman and one on the FTIR-spectra) was calibrated to classify the two C-sources (phenanthrene or glucose).

In the absence of SPs or in the presence of sand, the metabolic profile of *P. putida* was comparable, regardless of the C-source (Figure 3). A strong effect of phenanthrene on *P. putida* metabolic profile was therefore excluded, which could be due to the fact that in our experiments cells were pre-acclimated to the use of phenanthrene and the necessary set of enzymes to metabolize it was already induced (Deveryshetty and Phale, 2009). Moreover, Vandera et al. (2015) found that half of the identified proteins in *Arthrobacter phenanthrenivorans* Sphe3 are shared between phenanthrene and glucose-grown cells and only a small pool of the whole proteome is up or down-regulated at the two growth conditions. It is therefore evident that the metabolic response to phenanthrene is limited to very specific macromolecular targets and that *P. putida* possesses a very effective array of de-toxifying and protective mechanisms against phenanthrene (Domínguez-Cuevas et al., 2006; Vandera et al., 2015).

Interestingly, the effect of phenanthrene, relative to glucose, was unambiguously evident when cells were grown in the presence of clays or humic acids (Figure 3). The metabolic changes must have been triggered by a modified accessibility to the C-source. This finding is further supported by the fact that clays and humic acids slightly altered the phenanthrene solubility in our cultures. Furthermore, soil components such as clays and humic acids, adsorb PAHs (Müller et al., 2007; He and Wang, 2011) thereby altering the physico-chemical processes occurring at the exchange surface between bacterial cells and the surrounding environment. Clays can create favorable micro-environment where cells are found in close proximity to C and other nutrient sources (Filip, 1973), and this is particularly true in the presence of PAHs (Ortega-Calvo and Saiz-Jimenez, 1998). Humic acids work as surfactants that can facilitate the transport of phenanthrene across cell membranes helping its acquisition (Xie et al., 2017). Also, it cannot be excluded that basal macromolecular divergences between planktonic and sessile cells (Vilain et al., 2004; Ammons et al., 2014; Favre et al., 2017) made more prominent the effect of the two C-sources (Figure 3). In any case, by creating micro-niches with different physico-chemical properties, the SPs induced *P. putida* to express different final metabolic profiles as a function of the C-source. This reveals for the first time the effect of SPs on bacteria metabolic profile during PAHs biodegradation. We have to point out that since we did not analyze single cells, we can not confirm that the effect of the SPs on the metabolic profile of the cells was exclusively related to the adhesion of the cells to the particles or to other indirect effects induced by the presence of the particles.

Rather than a strong quantitative reorganization of targeted C-pools, *P. putida* underwent a general adjustment of the overall macromolecular profile (Supplementary Figure S2). Probably, this reorganization was enacted in the attempt to improve the binding to mineral particles, to adjust enzyme expression or the release of surfactants finalized at optimizing the capture and assimilation of phenanthrene molecules. The most prominent loadings indicated that the difference between the glucose and phenanthrene cell metabolic profiles was due to different levels of proteins, amino acids, nucleic acids and carbohydrates. Vandera et al. (2015) found that several cell functions (e.g., specific membrane transporters, PAHs degrading enzymes and the aromatic amino acids degradation pathways) were up-regulated, whereas the synthesis of structural features (i.e., peptidoglycan) was down-regulated when the cells were consuming phenanthrene with respect to glucose. Xie et al. (2017) also reported the adjustments of *Sphingobium* sp. cell wall properties grown in the presence of humic acids and phenanthrene. These changes are consistent with the loadings identified by our model, which suggest a reorganization of the proteome to optimize phenanthrene acquisition as well as structural changes at the cell surface (Keum et al., 2008; Seo et al., 2009).

Overall, our results reveal that the spatial and compositional (mineral and organic components) heterogeneity of soil affect, beside bacterial diversity (Kuzyakov and Blagodatskaya, 2015), also the variety of metabolic profiles expressed at the single species level. A direct consequence of this finding is that distinct cell metabolic profiles induced by the presence of determinate SPs can reflect cells with different functions, even at the single species level. This may define new limits to the definition of ecological functions and roles that we already know, especially for what it concerns PAHs degradation.

As a first attempt to investigate the effect of different soil components on the metabolic profile of bacteria, we have to point out that further research might be necessary to extend and proof these conclusions to real soil samples where mineral and organic components are in close contact the one with the others and are present in mixture with different ratios. Finally, a higher instrumental spatial resolution would allow to acquire spectra of single cells and to more precisely resolve the metabolic differences among planktonic and adhered cells and therefore disentangle the direct from the indirect effects of SPs on cell metabolic profile.

## Data Availability Statement

The raw data supporting the conclusions of this manuscript will be made available by the authors, without undue reservation, to any qualified researcher.

## Author Contributions

AF performed the experiments, analyzed the results, and wrote the paper. AZ, CM, and AC conceived the study, directed research, analyzed the results, and contributed to the manuscript writing. AC is the PI of the RhizOrg project that funded this study.

## Conflict of Interest Statement

The authors declare that the research was conducted in the absence of any commercial or financial relationships that could be construed as a potential conflict of interest.

## References

[B1] Amellal N. Portal J.-M. Vogel T. Berthelin J. (2001). Distribution and location of polycyclic aromatic hydrocarbons (PAHs) and PAH-degrading bacteria within polluted soil aggregates. *Biodegradation* 12 49–57. 10.1023/A:1011909107858 11693295

[B2] Ammons M. C. B. Tripet B. P. Carlson R. P. Kirker K. R. Gross M. A. Stanisich J. J. (2014). Quantitative NMR metabolite profiling of methicillin-resistant and methicillin-susceptible *Staphylococcus aureus* discriminates between biofilm and planktonic phenotypes. *J. Proteome Res.* 13 2973–2985. 10.1021/pr500120c 24809402 PMC4059261

[B3] Andrews J. S. Rolfe S. A. Huang W. E. Scholes J. D. Banwart S. A. (2010). Biofilm formation in environmental bacteria is influenced by different macromolecules depending on genus and species. *Environ. Microbiol.* 12 2496–2507. 10.1111/j.1462-2920.2010.02223.x 20406292

[B4] Barker M. Rayens W. (2003). Partial least squares for discrimination. *J. Chemom.* 17 166–173. 10.1002/cem.785

[B5] Barnes R. J. Dhanoa M. S. Lister S. J. (1989). Standard normal variate transformation and de-trending of near-infrared diffuse reflectance spectra. *Appl. Spectrosc.* 43 772–777. 10.1366/0003702894202201

[B6] Barth A. (2007). Infrared spectroscopy of proteins. *Biochim. Biophys. Acta* 1767 1073–1101. 10.1016/j.bbabio.2007.06.004 17692815

[B7] Beech I. Hanjagsit L. Kalaji M. Neal A. L. Zinkevich V. (1999). Chemical and structural characterization of exopolymers produced by *Pseudomonas sp*. NCIMB 2021 in continuous culture. *Microbiol. Read. Engl.* 145(Pt 6), 1491–1497. 10.1099/13500872-145-6-1491 10411276

[B8] Bushnell L. D. Haas H. F. (1941). The utilization of certain hydrocarbons by microorganisms. *J. Bacteriol.* 41 653–673. 16560430 10.1128/jb.41.5.653-673.1941 PMC374727

[B9] Cébron A. Faure P. Lorgeoux C. Ouvrard S. Leyval C. (2013). Experimental increase in availability of a PAH complex organic contamination from an aged contaminated soil: consequences on biodegradation. *Environ. Pollut.* 177 98–105. 10.1016/j.envpol.2013.01.043 23500046

[B10] Cébron A. Norini M.-P. Beguiristain T. Leyval C. (2008). Real-Time PCR quantification of PAH-ring hydroxylating dioxygenase (PAH-RHDα) genes from Gram positive and Gram negative bacteria in soil and sediment samples. *J. Microbiol. Methods* 73 148–159. 10.1016/j.mimet.2008.01.009 18329116

[B11] Chenu C. (1993). Clay- or sand-polysaccharide associations as models for the interface between micro-organisms and soil: water related properties and microstructure. *Geoderma* 56 143–156. 10.1016/0016-7061(93)90106-U

[B12] Deveryshetty J. Phale P. S. (2009). Biodegradation of phenanthrene by *Pseudomonas sp*. strain PPD: purification and characterization of 1-hydroxy-2-naphthoic acid dioxygenase. *Microbiol. Read. Engl.* 155 3083–3091. 10.1099/mic.0.030460-0 19574301

[B13] Domínguez-Cuevas P. González-Pastor J.-E. Marqués S. Ramos J.-L. de Lorenzo V. (2006). Transcriptional tradeoff between metabolic and stress-response programs in *Pseudomonas putida* KT2440 cells exposed to toluene. *J. Biol. Chem.* 281 11981–11991. 10.1074/jbc.M509848200 16495222

[B14] Fanesi A. Wagner H. Wilhelm C. (2017). Phytoplankton growth rate modelling: can spectroscopic cell chemotyping be superior to physiological predictors? *Proc. Biol. Sci. U.S.A* 284:20161956. 10.1098/rspb.2016.1956 28148743 PMC5310597

[B15] Favre L. Ortalo-Magné A. Greff S. Pérez T. Thomas O. P. Martin J.-C. (2017). Discrimination of four marine biofilm-forming bacteria by LC-MS metabolomics and cnfluence of Culture parameters. *J. Proteome Res.* 16 1962–1975. 10.1021/acs.jproteome.6b01027 28362105

[B16] Filip Z. (1973). Clay minerals as a factor influencing the biochemical activity of soil microorganisms. *Folia Microbiol.* 18 56–74. 10.1007/BF02884250 4687409

[B17] Fonville J. M. Richards S. E. Barton R. H. Boulange C. L. Ebbels T. M. D. Nicholson J. K. (2010). The evolution of partial least squares models and related chemometric approaches in metabonomics and metabolic phenotyping. *J. Chemom.* 24 636–649. 10.1002/cem.1359

[B18] Haritash A. K. Kaushik C. P. (2009). Biodegradation aspects of polycyclic aromatic hydrocarbons (PAHs): a review. *J. Hazard. Mater.* 169 1–15. 10.1016/j.jhazmat.2009.03.137 19442441

[B19] He Y. Y. Wang X. C. (2011). Adsorption of a typical polycyclic aromatic hydrocarbon by humic substances in water and the effect of coexisting metal ions. *Colloids Surf. Physicochem. Eng. Asp.* 379 93–101. 10.1016/j.colsurfa.2010.12.023

[B20] Huang W. E. Li M. Jarvis R. M. Goodacre R. Banwart S. A. (2010). Shining light on the microbial world: the application of Raman microspectroscopy. *Adv. Appl. Microbiol.* 70 153–186. 10.1016/S0065-2164(10)70005-8 20359457

[B21] Huang W. E. Stoecker K. Griffiths R. Newbold L. Daims H. Whiteley A. S. (2007). Raman-FISH: combining stable-isotope Raman spectroscopy and fluorescence *in situ* hybridization for the single cell analysis of identity and function. *Environ. Microbiol.* 9 1878–1889. 10.1111/j.1462-2920.2007.01352.x 17635536

[B22] Indahl U. G. Liland K. H. Næs T. (2009). Canonical partial least squares—a unified PLS approach to classification and regression problems. *J. Chemom.* 23 495–504. 10.1002/cem.1243

[B23] Jiang W. Saxena A. Song B. Ward B. B. Beveridge T. J. Myneni S. C. B. (2004). Elucidation of functional groups on gram-positive and gram-negative bacterial surfaces using infrared spectroscopy. *Langmuir ACS J. Surf. Colloids* 20 11433–11442. 10.1021/la049043 15595767

[B24] Kachlany S. C. Levery S. B. Kim J. S. Reuhs B. L. Lion L. W. Ghiorse W. C. (2001). Structure and carbohydrate analysis of the exopolysaccharide capsule of *Pseudomonas putida* G7. *Environ. Microbiol.* 3 774–784. 10.1046/j.1462-2920.2001.00248.x 11846771

[B25] Kahm M. Hasenbrink G. Lichtenberg-Fraté H. Ludwig J. Kschischo M. (2010). grofit: fitting biological growth curves with R. *J. Stat. Softw.* 33 1–21. 10.18637/jss.v033.i0720808728

[B26] Kanti Sen T. Khilar K. C. (2006). Review on subsurface colloids and colloid-associated contaminant transport in saturated porous media. *Adv. Colloid Interface Sci.* 119 71–96. 10.1016/j.cis.2005.09.001 16324681

[B27] Keum Y. S. Lee Y. J. Kim J.-H. (2008). Metabolism of nitrodiphenyl ether herbicides by dioxin-degrading bacterium *Sphingomonas wittichii* RW1. *J. Agric. Food Chem.* 56 9146–9151. 10.1021/jf801362k 18778066

[B28] Kuhn M. (2017). *Caret: Classification and Regression Training. R Package Version 6.0-78*. Available at: https://cran.r-project.org/web/packages/caret/index.html

[B29] Kuzyakov Y. Blagodatskaya E. (2015). Microbial hotspots and hot moments in soil: concept & review. *Soil Biol. Biochem.* 83 184–199. 10.1016/j.soilbio.2015.01.025

[B30] LaRowe D. E. Amend J. P. (2016). The energetics of anabolism in natural settings. *ISME J.* 10 1285–1295. 10.1038/ismej.2015.227 26859771 PMC5029197

[B31] Lerch T. Z. Chenu C. Dignac M. F. Barriuso E. Mariotti A. (2017). Biofilm vs. Planktonic Lifestyle: consequences for Pesticide 2,4-D metabolism by *Cupriavidus necator* JMP134. *Front. Microbiol.* 8:904. 10.3389/fmicb.2017.00904 28588567 PMC5440565

[B32] Liland K. H. Mevik B. H. Canteri R. (2015). *Baseline: Baseline Correction of Spectra. R Package Version, 1.2-1*. Available at: https://cran.r-project.org/web/packages/baseline/index.html

[B33] Lima-Morales D. Jáuregui R. Camarinha-Silva A. Geffers R. Pieper D. H. Vilchez-Vargas R. (2016). Linking microbial community and catabolic gene structures during the adaptation of three contaminated soils under continuous long term pollutant stress. *Appl. Environ. Microbiol.* 82 2227–2237. 10.1128/AEM.03482-15 26850298 PMC4807512

[B34] Louvel B. Cébron A. Leyval C. (2011). Root exudates affect phenanthrene biodegradation, bacterial community and functional gene expression in sand microcosms. *Int. Biodeterior. Biodegrad.* 65 947–953. 10.1016/j.ibiod.2011.07.003

[B35] McCarty P. L. (2007). Thermodynamic electron equivalents model for bacterial yield prediction: modifications and comparative evaluations. *Biotechnol. Bioeng.* 97 377–388. 10.1002/bit.21250 17089390

[B36] Mevik B. H. Wehrens R. Liland K. H. (2016). *Pls: Partial Least Squares and Principal Component Regression. R package version 2.6-0*. Available at: https://cran.r-project.org/web/packages/pls/index.html

[B37] Miyata N. Iwahori K. Foght J. M. Gray M. R. (2004). Saturable, energy-dependent uptake of phenanthrene in aqueous phase by *Mycobacterium sp*. strain RJGII-135. *Appl. Environ. Microbiol.* 70 363–369. 10.1128/AEM.70.1.363-369.2004 14711664 PMC321281

[B38] Mohr S. C. Sokolov N. V. He C. M. Setlow P. (1991). Binding of small acid-soluble spore proteins from *Bacillus subtilis* changes the conformation of DNA from B to A. *Proc. Natl. Acad. Sci. U.S.A.* 88 77–81. 10.1073/pnas.88.1.77 1898779 PMC50751

[B39] Moreno-Forero S. K. Van Der Meer J. R. (2015). Genome-wide analysis of *Sphingomonas wittichii* RW1 behavior during inoculation and growth in contaminated sand. *ISME J.* 9 150–165. 10.1038/ismej.2014.101 24936762 PMC4274413

[B40] Movasaghi Z. Rehman S. Rehman I. U. (2007). Raman spectroscopy of biological tissues. *Appl. Spectrosc. Rev.* 42 493–541. 10.1080/05704920701551530

[B41] Movasaghi Z. Rehman S. Rehman I. U. (2008). Fourier Transform Infrared (FTIR) spectroscopy of biological tissues. *Appl. Spectrosc. Rev.* 43 134–179. 10.1080/05704920701829043

[B42] Müller S. Totsche K. U. Kögel-Knabner I. (2007). Sorption of polycyclic aromatic hydrocarbons to mineral surfaces. *Eur. J. Soil Sci.* 58 918–931. 10.1111/j.1365-2389.2007.00930.x

[B43] Naumann D. (2001). FT-Infrared and FT-Raman spectroscopy in biomedical research. *Appl. Spectrosc. Rev.* 36 239–298. 10.1081/ASR-100106157 23792287

[B44] Neugebauer U. Schmid U. Baumann K. Ziebuhr W. Kozitskaya S. Deckert V. (2007). Towards a detailed understanding of bacterial metabolism—spectroscopic characterization of *Staphylococcus Epidermidis*. *ChemPhysChem* 8 124–137. 10.1002/cphc.200600507 17146809

[B45] Ojeda J. J. Romero-Gonzalez M. E. Pouran H. M. Banwart S. A. (2008). *In situ* monitoring of the biofilm formation of *Pseudomonas putida* on hematite using flow-cell ATR-FTIR spectroscopy to investigate the formation of inner-sphere bonds between the bacteria and the mineral. *Mineral. Mag.* 72 101–106. 10.1180/minmag.2008.072.1.101

[B46] Ortega-Calvo J.-J. Saiz-Jimenez C. (1998). Effect of humic fractions and clay on biodegradation of phenanthrene by a *Pseudomonas fluorescens* strain isolated from soil. *Appl. Environ. Microbiol.* 64 3123–3126. 9687489 10.1128/aem.64.8.3123-3126.1998 PMC106831

[B47] Parikh S. J. Chorover J. (2006). ATR-FTIR spectroscopy reveals bond formation during bacterial adhesion to iron oxide. *Langmuir* 22 8492–8500. 10.1021/la061359p 16981768

[B48] R Development Core Team (2010). *R: A Language and Environment for Statistical Computing.* Vienna: The R Project for Statistical Computing.

[B49] Rygula A. Majzner K. Marzec K. M. Kaczor A. Pilarczyk M. Baranska M. (2013). Raman spectroscopy of proteins: a review. *J. Raman Spectrosc.* 44 1061–1076. 10.1002/jrs.4335

[B50] Sackett O. Petrou K. Reedy B. Grazia A. D. Hill R. Doblin M. (2013). Phenotypic plasticity of Southern Ocean diatoms: key to success in the sea ice habitat? *PLoS One* 8:e81185. 10.1371/journal.pone.0081185 24363795 PMC3868450

[B51] Savitzky A. Golay M. J. E. (1964). Smoothing and differentiation of data by simplified least squares procedures. *Anal. Chem.* 36 1627–1639. 10.1021/ac60214a047 21322220

[B52] Seo J.-S. Keum Y.-S. Li Q. X. (2009). Bacterial degradation of aromatic compounds. *Int. J. Environ. Res. Public. Health* 6 278–309. 10.3390/ijerph6010278 19440284 PMC2672333

[B53] Tecon R. Wells M. Van Der Meer J. R. (2006). A new green fluorescent protein-based bacterial biosensor for analysing phenanthrene fluxes. *Environ. Microbiol.* 8 697–708. 10.1111/j.1462-2920.2005.00948.x 16584481

[B54] Teng L. Wang X. Wang X. Gou H. Ren L. Wang T. (2016). Label-free, rapid and quantitative phenotyping of stress response in *E. coli* via ramanome. *Sci. Rep.* 6:sre34359. 10.1038/srep34359 27756907 PMC5069462

[B55] Thomas F. Lorgeoux C. Faure P. Billet D. Cébron A. (2016). Isolation and substrate screening of polycyclic aromatic hydrocarbon degrading bacteria from soil with long history of contamination. *Int. Biodeterior. Biodegrad.* 107 1–9. 10.1016/j.ibiod.2015.11.004

[B56] Uyttebroek M. Breugelmans P. Janssen M. Wattiau P. Joffe B. Karlson U. (2006). Distribution of the *Mycobacterium* community and polycyclic aromatic hydrocarbons (PAHs) among different size fractions of a long-term PAH-contaminated soil. *Environ. Microbiol.* 8 836–847. 10.1111/j.1462-2920.2005.00970.x 16623741

[B57] VanBriesen J. M. (2001). Thermodynamic yield predictions for biodegradation through oxygenase activation reactions. *Biodegradation* 12 263–279. 10.1023/A:1013179315518 11826909

[B58] Vandera E. Samiotaki M. Parapouli M. Panayotou G. Koukkou A. I. (2015). Comparative proteomic analysis of *Arthrobacter phenanthrenivorans* Sphe3 on phenanthrene, phthalate and glucose. *J. Proteomics* 113 73–89. 10.1016/j.jprot.2014.08.018 25257624

[B59] Vandevivere P. Kirchman D. L. (1993). Attachment stimulates exopolysaccharide synthesis by a bacterium. *Appl. Environ. Microbiol.* 59 3280–3286. 16349064 10.1128/aem.59.10.3280-3286.1993 PMC182449

[B60] Vilain S. Cosette P. Hubert M. Lange C. Junter G.-A. Jouenne T. (2004). Comparative proteomic analysis of planktonic and immobilized *Pseudomonas aeruginosa* cells: a multivariate statistical approach. *Anal. Biochem.* 329 120–130. 10.1016/j.ab.2004.02.014 15136174

[B61] Wagner H. Jungandreas A. Fanesi A. Wilhelm C. (2014). Surveillance of C-allocation in microalgal cells. *Metabolites* 4 453–464. 10.3390/metabo4020453 24957036 PMC4101516

[B62] Wagner H. Liu Z. Langner U. Stehfest K. Wilhelm C. (2010). The use of FTIR spectroscopy to assess quantitative changes in the biochemical composition of microalgae. *J. Biophotonics* 3 557–566. 10.1002/jbio.201000019 20503222

[B63] Wagner M. (2009). Single-cell ecophysiology of microbes as revealed by Raman microspectroscopy or secondary ion mass spectrometry imaging. *Annu. Rev.Microbiol.* 63 411–429. 10.1146/annurev.micro.091208.073233 19514853

[B64] Weissenfels W. D. Klewer H.-J. Langhoff J. (1992). Adsorption of polycyclic aromatic hydrocarbons (PAHs) by soil particles: influence on biodegradability and biotoxicity. *Appl. Microbiol. Biotechnol.* 36 689–696. 10.1007/BF00183251 1368071

[B65] Whelan D. R. Bambery K. R. Heraud P. Tobin M. J. Diem M. McNaughton D. (2011). Monitoring the reversible B to A-like transition of DNA in eukaryotic cells using Fourier transform infrared spectroscopy. *Nucleic Acids Res.* 39 5439–5448. 10.1093/nar/gkr175 21447564 PMC3141270

[B66] Whelan D. R. Hiscox T. J. Rood J. I. Bambery K. R. McNaughton D. Wood B. R. (2014). Detection of an en masse and reversible B- to A-DNA conformational transition in prokaryotes in response to desiccation. *J. R. Soc. Interface* 11:20140454. 10.1098/rsif.2014.0454 24898023 PMC4208382

[B67] Wick L. Y. Colangelo T. Harms H. (2001). Kinetics of mass transfer-limited bacterial growth on solid PAHs. *Environ. Sci. Technol.* 35 354–361. 10.1021/es001384w 11347609

[B68] Wick L. Y. Pelz O. Bernasconi S. M. Andersen N. Harms H. (2003). Influence of the growth substrate on ester-linked phospho- and glycolipid fatty acids of PAH-degrading *Mycobacterium sp*. LB501T. *Environ. Microbiol.* 5 672–680. 10.1046/j.1462-2920.2003.00455.x 12871234

[B69] Wilcke W. Zech W. Kobža J. (1996). PAH-pools in soils along a PAH-deposition gradient. *Environ. Pollut.* 92 307–313. 10.1016/0269-7491(95)00110-7 15091383

[B70] Wold S. Sjöström M. Eriksson L. (2001). PLS-regression: a basic tool of chemometrics. *Chemom. Intell. Lab. Syst.* 58 109–130. 10.1016/S0169-7439(01)00155-1

[B71] Wu H. Chen W. Rong X. Cai P. Dai K. Huang Q. (2014a). Adhesion of *Pseudomonas putida* onto kaolinite at different growth phases. *Chem. Geol.* 390 1–8. 10.1016/j.chemgeo.2014.10.008

[B72] Wu H. Chen W. Rong X. Cai P. Dai K. Huang Q. (2014b). In situ ATR-FTIR study on the adhesion of *Pseudomonas putida* to Red soil colloids. *J. Soils Sediments* 14 504–514. 10.1007/s11368-013-0817-9

[B73] Wu H. Chen W. Rong X. Cai P. Dai K. Huang Q. (2014c). Soil colloids and minerals modulate metabolic activity of *Pseudomonas putida* measured using microcalorimetry. *Geomicrobiol. J.* 31 590–596. 10.1080/01490451.2013.861544

[B74] Xie Y. Gu Z. Herath H. M. S. K. Gu M. He C. Wang F. (2017). Evaluation of bacterial biodegradation and accumulation of phenanthrene in the presence of humic acid. *Chemosphere* 184 482–488. 10.1016/j.chemosphere.2017.06.026 28618280

