# Supplementary material for: Soil Particles and Phenanthrene Interact in Defining the Metabolic Profile of Pseudomonas putida G7: A Vibrational Spectroscopy Approach
Source: Front Microbiol. 2018 Dec 4;9:2999. doi: 10.3389/fmicb.2018.02999 (PMC6288191; doi:10.3389/fmicb.2018.02999)
Supplement: Supplementary file 2 [file Image_2.pdf]

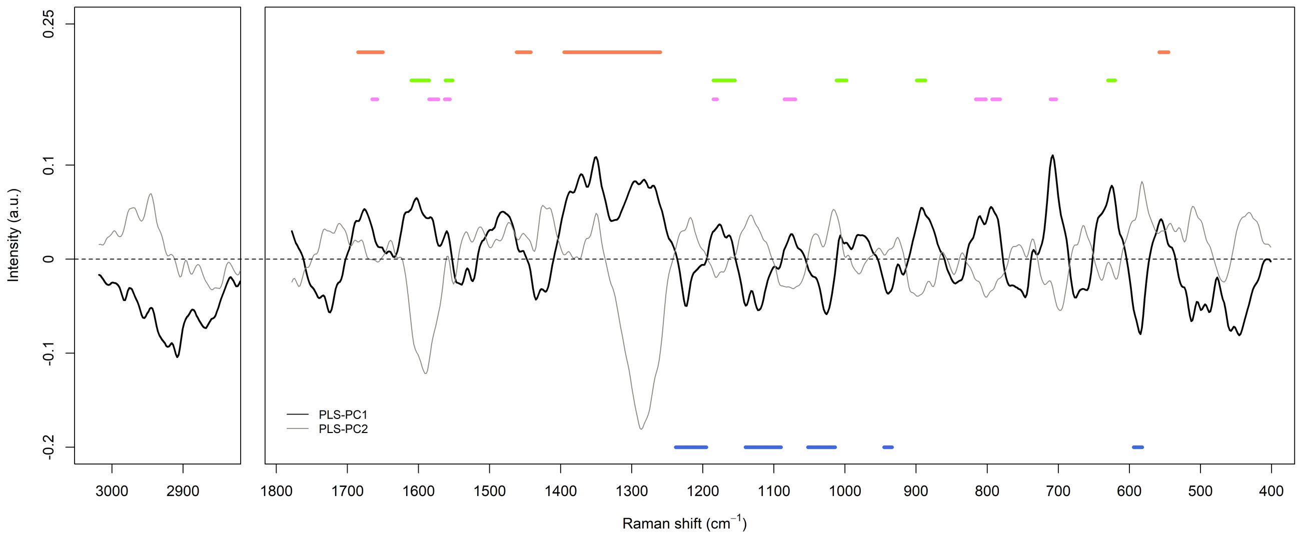

Figure S2. Loadings plot of the model discriminating for the C-source (glucose vs. phenanthrene) based on FT-Raman spectra. Horizontal bold lines in the loading plots represent the macromolecular assignment of the peaks (— proteins, — nucleic acids, — amino acids and — carbohydrates). The sign (-/+) of the bold lines reflect that of the corresponding loading of the PLS-PC1.
