# Supplementary material for: Soil Particles and Phenanthrene Interact in Defining the Metabolic Profile of Pseudomonas putida G7: A Vibrational Spectroscopy Approach
Source: Front Microbiol. 2018 Dec 4;9:2999. doi: 10.3389/fmicb.2018.02999 (PMC6288191; doi:10.3389/fmicb.2018.02999)
Supplement: Supplementary file 3 [file Image_3.pdf]

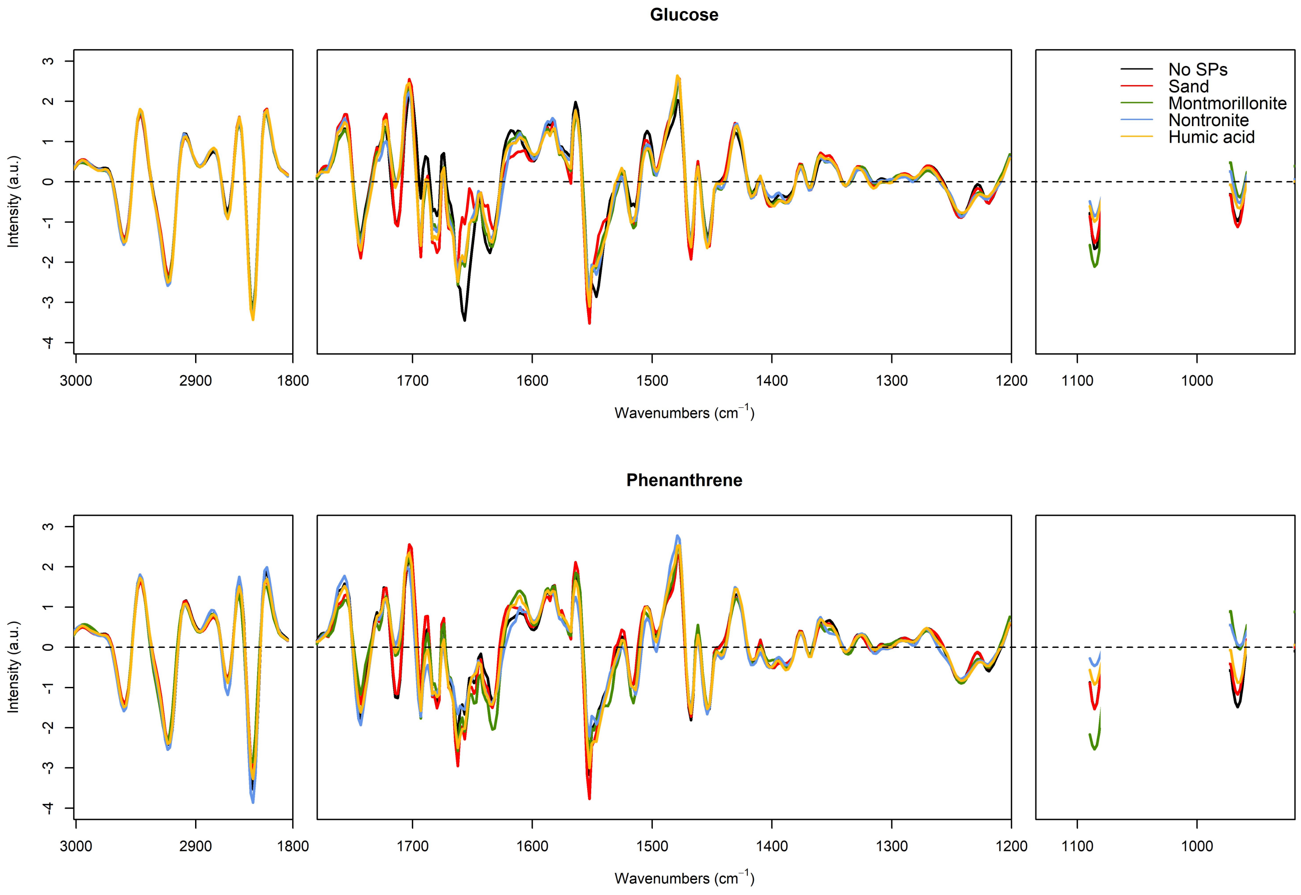

Figure S3. Average FTIR spectra of *P. putida* grown in the absence and presence of different SPs and metabolizing glucose (upper panel) or phenanthrene (lower panel) as the sole energy and C-source. The spectra were converted to 2nd derivatives and normalized using the standard normal variate transformation (see main text).
