# Supplementary material for: Soil Particles and Phenanthrene Interact in Defining the Metabolic Profile of Pseudomonas putida G7: A Vibrational Spectroscopy Approach
Source: Front Microbiol. 2018 Dec 4;9:2999. doi: 10.3389/fmicb.2018.02999 (PMC6288191; doi:10.3389/fmicb.2018.02999)
Supplement: Supplementary file 4 [file Image_4.pdf]

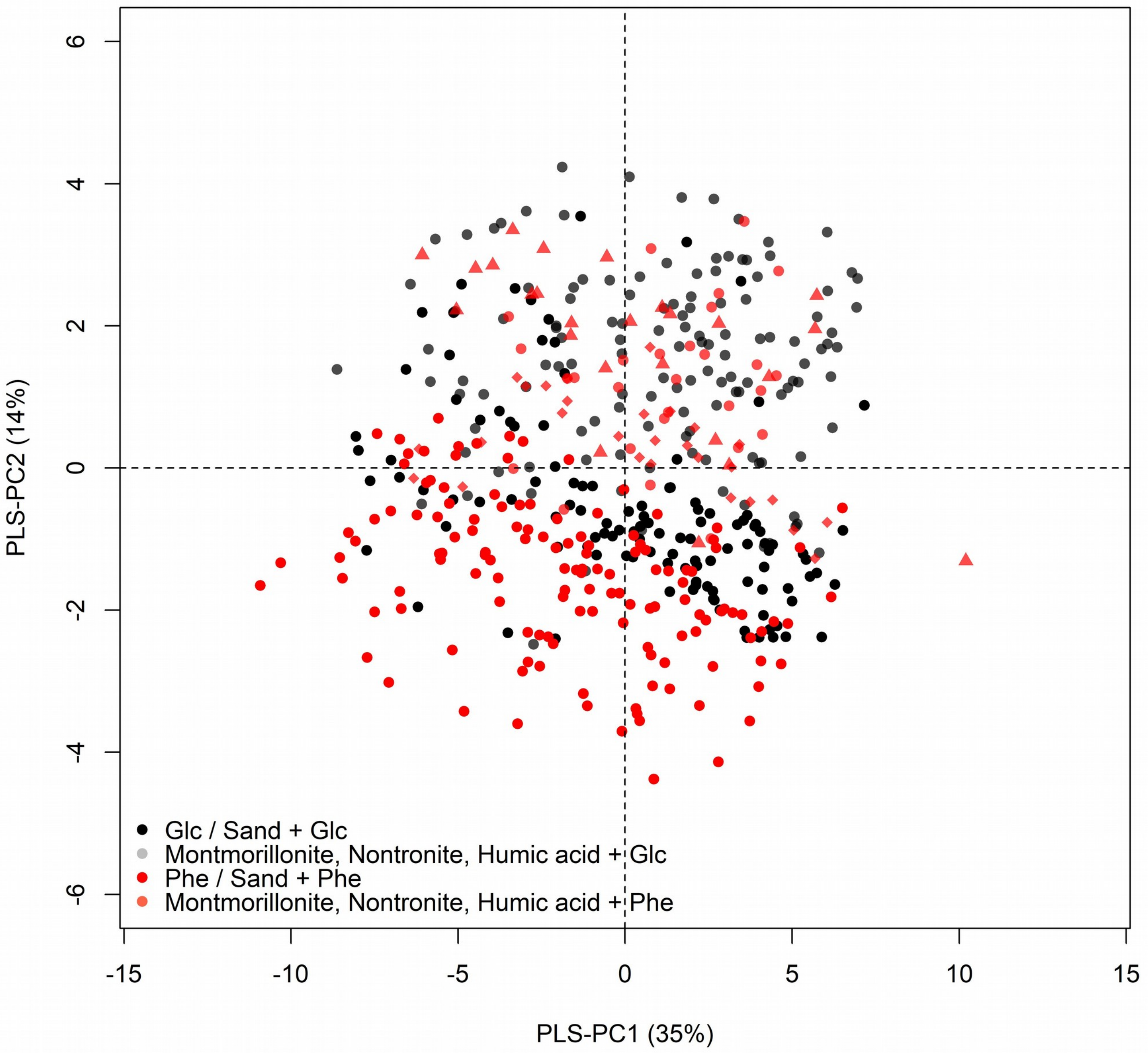

Figure S4. Partial Least Square Discriminant analysis (PLS-DA). Scores plot of the model calibrated to discriminate the C-source (phenanthrene (Phe) and glucose (Glc)) based on FTIR spectra.
