# Supplementary material for: Soil Particles and Phenanthrene Interact in Defining the Metabolic Profile of Pseudomonas putida G7: A Vibrational Spectroscopy Approach
Source: Front Microbiol. 2018 Dec 4;9:2999. doi: 10.3389/fmicb.2018.02999 (PMC6288191; doi:10.3389/fmicb.2018.02999)
Supplement: Supplementary file 6 [file Table_1.pdf]

Table S1. Variance explained by the first eight principal components (PLS-PC) of the PLS-DA model calibrated for the discrimination of each experimental treatment (i.e. ± SPs and glucose (Glc) or phenanthrene (Phe)) based on FT-Raman spectra of *Pseudomonas putida*. The number of components for each model was chosen based on the minimal RMSEP and maximal R^2 values.

	**PLS-PC1**	**PLS-PC2**	**PLS-PC3**	**PLS-PC4**	**PLS-PC5**	**PLS-PC6**	**PLS-PC7**	**PLS-PC8**
**FT-Raman**	10.60	6.49	8.64	2.87	1.45	1.34	4.04	1.64
**FTIR**	9.36	3.92	4.75	2.28	3.07	0.94	8.73	5.26
