# Supplementary material for: Soil Particles and Phenanthrene Interact in Defining the Metabolic Profile of Pseudomonas putida G7: A Vibrational Spectroscopy Approach
Source: Front Microbiol. 2018 Dec 4;9:2999. doi: 10.3389/fmicb.2018.02999 (PMC6288191; doi:10.3389/fmicb.2018.02999)
Supplement: Supplementary file 7 [file Table_2.pdf]

Table S2. Band assignment, sign and macromolecular origins of FT-Raman and FTIR PLS-DA loadings. Band assignment is based on literature references (Huang et al., 2010; Movasaghi et al., 2007a, 2008a; Naumann, 2001b) and on the reference spectra of pure macromolecules.

FT-Raman

PLS-PC1	Sign	PLS-PC2	Sign	Tentative band identification	Macromolecular assignment
2956	-			CH_3 asymmetric stretching	Lipids
2921	-			CH band	Proteins, Lipids and nucleic acids
	-	2906		CH stretch	Carbohydrates
2867		2865		CH_2 symmetric stretch of lipids and CH_2 asymmetric stretch of proteins and lipids	Proteins and lipids
1675	+			C=O symmetric stretching Amide I (β-sheets)	Proteins
		1662	-	Amide I, DNA NH deformation	Proteins and nucleic acids
1600	+			Phenylalanine (C=CH deformation), tyrosine	Amino acids,
		1591	-	Phenylalanine	Amino acids
1581	+	1575	-	G, A, ring breathing mode in nucleic acid bases	Nucleic acids
1558	+	1560	+	Ring breathing mode in tryptophan, and nucleic acids	Amino acids, Nucleic acids
1452	+	/		CH_2 bending	Proteins and lipids
1419	-	1425	+	Symmetric deformation of COO-	Proteins and lipids
1328-1395 max (1350)	+	1348	+	CH deformation	Proteins and carbohydrates
1238-1328 max (1297)	+	1278	-	Amide III	Proteins
1195-1238 max (1222)	-	1216	+	carb (from our reference spectra)	Carbohydrates
		1182	-	C, G, A ring breathing mode	Nucleic acids
1174/1162	+			CH deformation in phenylalanine and tyrosine,	Amino acids
1090-1140 max (1093, 1118, 1139)	-	1089	-	CO and CC stretching	Carbohydrates
1018-1050 max (1027)	-			CO, CC and C-OH symmetric stretching	Carbohydrates
1076	+	1068	-	sym. PO_2- stretching of DNA	Nucleic acids
1006	+	1016	-	CC symmetric ring breathing mode of phenylalanine	Amino acids
939	-			C-O-C	Carbohydrates
891	+	887	-	Tryptophan δ(ring)	Amino acids
811	+	802	-	O-P-O- stretch in RNA	Nucleic acids
788	+			CC ring stretching of cytosine and uracyl	Nucleic acids
707	+	707	+	CC ring stretching of adenine	Nucleic acids
626	+	636, 619	-	CC twisting mode of phenylalanine	Amino acids
586	-	582	+	carbohydrates	Carbohydrates
555	+	551	-	S-S stretching	Proteins

FTIR

PLS-PC1	Sign	PLS-PC2	Sign	Tentative band identification	Macromolecular assignment
2964	-	2964	-	asymmetric stretching of CH from CH_3	Proteins and lipids
2917	+	2917	-	CH asymmetric stretching of CH_2 in fatty acids	lipids
2873	-	2873	-	CH symmetric stretching of CH_3	Proteins
2848	+	2850	-	CH symmetric stretching of CH_2 in fatty acids	Lipids
1743	+	1747	-	C=O stretching mode of esters in triglycerides and fatty acids	Lipids
1712	+	1710	+	C=O stretching of nucleic acid bases	Nucleic acids
1695	-	1697	-	A high frequency vibration of an antiparallel β-sheets of AmideI	Proteins
1675	-	1673	-	β-turns of proteins	Proteins
1656	+	1654	+	α helical structure of Amide I	Proteins
1629	-	1621		β-sheet structure of Amide I	Proteins
		1555	-	Ring base	Nucleic acids
1544	+	/		Amide II (dN-H, nC-N)	Proteins
1452	/	1455	-	Asymmetric methyl deformation	Proteins
1410	+			v(C=C) aromatic aminoacids	Proteins
1245	+	1245	+	P=O asymmetric stretching of PO_2^- phosphodiester	Nucleic acids
1230	-	1228	-	P=O asymmetric stretching of PO_2^- phosphodiester	Nucleic acids
1093	-	1083	+	P=O symmetric stretching of PO_2^-	Nucleic acids
968	+	962	-	backbone C-C stretching in DNA	Nucleic acids
