# Supplementary material for: Soil Particles and Phenanthrene Interact in Defining the Metabolic Profile of Pseudomonas putida G7: A Vibrational Spectroscopy Approach
Source: Front Microbiol. 2018 Dec 4;9:2999. doi: 10.3389/fmicb.2018.02999 (PMC6288191; doi:10.3389/fmicb.2018.02999)
Supplement: Supplementary file 8 [file Table_3.pdf]

**Table S3.** Diagnostic parameters of the PLS-DA models discriminating for the C-source (phenanthrene or glucose). The number of components for each model was chosen based on the minimal RMSEP and maximal $R^2$ values. For the FT-Raman model 4 principal components were used, whereas for the FTIR model 8 principal components were used. RMSC: root mean squared error of calibration, RMSEP: root mean squared error of prediction, $R^2_C$: coefficient of determination for the calibration, $R^2_P$: coefficient of determination for predicted values, Sensitivity: ability in discriminating true positives, Specificity: ability in discriminating true negatives.

| | **FT-Raman spectroscopy** | **FTIR spectroscopy** |
|---|---|---|
| **Sensitivity** | 0.82 | 0.92 |
| **Specificity** | 0.75 | 0.94 |
| **RMSEP** | 0.41 | 0.28 |
| **$R^2_p$** | 0.29 | 0.67 |
| **RMSEC** | 0.35 | 0.26 |
| **$R^2_c$** | 0.50 | 0.71 |
| **% variance X** | 38 | 87 |
| **% variance Y** | 50 | 71 |
